# Different Treatments in Patients with Temporomandibular Joint Disorders: A Comparative Randomized Study

**DOI:** 10.3390/medicina56030113

**Published:** 2020-03-05

**Authors:** Bruno Macedo De Sousa, Nansi López-Valverde, Antonio López-Valverde, Francisco Caramelo, Javier Flores Fraile, Julio Herrero Payo, María João Rodrigues

**Affiliations:** 1 Institute for Occlusion and Orofacial Pain Faculty of Medicine, University of Coimbra, Polo I-Edifício Central Rua Larga, 3004-504 Coimbra, Portugal; bsousa@fmed.uc.pt (B.M.D.S.); maria.jrodrigues@hotmail.com (M.J.R.); 2 Department of Surgery. University of Salamanca, Salamanca, Spain. Instituto de Investigación Biomédica de Salamanca (IBSAL), Avda. Alfonso X El Sabio S/N. 37007, Salamanca, Spain; anlopezvalverde@gmail.com (A.L.-V.);; 3 Laboratory of Biostatistics and Medical Informatics, Coimbra. Institute for Clinical and Biomedical Research (iCBR), School of Medicine, University of Coimbra, Polo 3, Azinhaga de Santa Comba, Celas 3000-548 Coimbra, Portugal

**Keywords:** temporomandibular disorders, arthralgia, bite splint, sodium hyaluronate, betamethasone, platelet-rich plasma

## Abstract

*Background and Objectives*: Temporomandibular joint disorders (TMJDs) are associated with pain and reduced jaw mobility. The aim of this study was to compare the outcome of patients with TMJ arthralgia when submitted to four different treatment modalities, in some cases using intra-articular injections of substances with anti-inflammatory properties and in others, a more conservative approach consisting only of a bite splint. *Materials and Methods*: The sample was made up of 80 patients, randomly distributed into 4 groups of 20 patients each. Each patient was given a nocturnal bite splint. One of the groups was treated with the bite splint only, while each patient in the other 3 was injected with betamethasone, sodium hyaluronate, or platelet-rich plasma in addition to using the bite splint. Two variables were assessed, namely pain intensity between 0 to 10 according to the visual analogue scale and maximum pain-free mouth opening in mm. The patients were evaluated at four different points: at the beginning of the treatment, as well as one week, one month and six months after initiation. *Results*: The results showed that maximum pain-free mouth opening improved in all the groups that made up the sample, with either a reduction in pain severity or with no pain. However, the group injected with platelet-rich plasma yielded the best results after six months, while patients treated with sodium hyaluronate or betamethasone obtained the best results at the end of the first week. *Conclusions*: We concluded that all the treatments used caused a reduction in pain and increased pain-free mouth opening. The splint combined with the platelet-rich plasma injection achieved long-term success.

## 1. Introduction

The term temporomandibular joint disorders (TMJDs) covers a broad spectrum of clinical issues related to joints and muscles in the orofacial area. These dysfunctions are mainly characterized by pain, joint sounds and irregular or limited jaw function. TMJDs are considered a distinct subgroup of musculoskeletal and rheumatoid disorders and represent the most common cause of orofacial pain of non-dental origin [1,2]. 

TMJDs are the most common chronic orofacial pain conditions and are often compared with cervical pain, back pain and headache. This is because of the intensity, persistence and fundamentally psychological impact on the patient of such pain. TMJDs are also known to be rare in children prior to puberty [3].

In the adult population, incidences of temporomandibular joint pain (TMJP) vary between 9% to 15% for women and 3% to 10% for men. TMJP seems to be 1.5 to 2 times more common in women than in men, although certain studies report a greater difference of up to 4.1. Similarly, in all studies where there was a clear age pattern, the age range where TMJP was more prevalent was between 35 and 45 years [4]. Studies on the incidence rate of TMJP indicate that it is about 2% to 3% per year [5,6,7]. This low incidence suggests that the high prevalence of pain in the population is due to its chronic condition.

The most common intraoral treatment for pain associated with TMJDs is the use of occlusal splints (bite splints), which are designed to cover the occlusive surfaces of the upper or lower teeth and thus reduce strain on the temporomandibular joint. There is a general consensus that the use of a bite splint should never promote permanent changes in teeth or jaw position [8,9,10]. Currently, the most widespread myorelaxant splint is the “Michigan splint”, developed in the 1960s by Ramfjord and Ash [11] with the following specifications: (i) always adjusted for maximum intercuspation, (ii) the inclination of the guide starts about 1 mm from the canine, (iii) there may be no incisal guide from occlusion at maximum intercuspation, (iv) allows the condyles to search for a position of maximum comfort and (v) can be used indefinitely without modifying dental occlusal relations.

On the other hand, intra-articular injections with corticosteroids have also been successfully used for different TMJ conditions, despite their potential adverse effects such as progression of a pre-existing joint lesion [12,13,14,15,16,17]. Corticosteroid therapy is usually combined with a local anesthetic, an association that, although controversial, is believed by certain authors to decrease the risk of complications compared to the isolated administration of corticosteroids [13,17,18,19].

Another compound used in TMJ intra-articular injections is sodium hyaluronate, used to either promote viscosity supplementation or act as an anti-inflammatory agent, which is currently reporting very promising results [14,16,20,21,22,23,24]. Hyaluronic acid is a polysaccharide of high molecular weight that is a physiological component of the synovial fluid responsible for joint lubrication. Synovial cells, fibroblasts and chondrocytes synthesize hyaluronic acid and secrete it inside the joint. Hyaluronic acid is primarily responsible for synovial fluid’s viscous and elastic nature. Thus, normal concentrations of hyaluronic acid act as a viscous lubricant in slow movements and as an elastic shock absorber in fast movements [25].

Platelet-rich plasma (PRP) is a therapeutic agent consisting essentially of a platelet concentrate and associated growth factors taken and centrifuged from a sample of the patient's blood. It was initially introduced in the fields of stomatology, maxillofacial/plastic surgery and reconstructive surgery in the 1990s and its clinical use is due to its potential healing properties through cell recruitment, proliferation, differentiation and consequently, tissue remodeling. It has been found to have several advantages over the use of corticosteroids in the treatment of TMJ degenerative and inflammatory conditions, the most remarkable being its lack of serious and/or irreversible adverse effects. Treatment with PRP injections has reported anti-inflammatory, analgesic and antibacterial properties and, at the same time, restores intra-articular levels of hyaluronic acid, increases glycosaminoglycan chondrocyte synthesis, balances joint angiogenesis and induces stem cell migration [26].

The aim of this study was to compare the outcome of patients with TMJ arthralgia regarding pain and mouth opening. Treatment consisted of a bite splint (control) or a bite splint in combination with corticoid (betamethasone) injections, sodium hyaluronate injections or PRP injections. This investigation took place in order to determine which treatment would be the most successful in improving the study variables.

## 2. Materials and Methods

### 2.1. Patients 

A sample of 80 patients diagnosed with TMJ arthralgia, according to the original version of the diagnostic criteria for temporomandibular disorders (DC/TMDs) were recruited from consultations framed in the Course of Occlusal Rehabilitation at the University of Coimbra, organized by the School of Medicine. All patients agreed to participate in the research and signed the informed consent form. This study was conducted in compliance with the Declaration of Helsinki and was approved by the ethics committee of the School of Medicine of the University of Coimbra (Coimbra, Portugal). This study was approved on 25 June 2017 by the institutional review board (IRB 06-2017-096).

### 2.2. Inclusion Criteria

Patients who met all the following requirements were included in the study: a clinical history of over 6 months of TMJP that modifies with mandibular movement in function or parafunction; pain present in a clinical examination at opening, laterality or palpation; and no previous treatment. Exclusion criteria: patients who had received previous treatment for TMJ dysfunction; patients suffering from any rheumatic pathology such as rheumatoid arthritis or psoriatic arthritis (including juvenile arthritis); hypnosis patients; pregnant or breastfeeding women; and those who were under 18 years old.

Patients were randomly assigned to four groups, each of them including 20 patients (*n* = 20): patients receiving bite splint therapy only (BS); patients using a bite splint and receiving betamethasone (BS + B); patients using a bite splint and treated with sodium hyaluronate (BS + SH); and patients using a bite splint and undergoing PRP treatment (BS + PRP).

### 2.3. Treatments

A bite splint was made for each patient, with contact points in all teeth and canine guidance in laterality and protrusion movements. The splints were worn during the night and the occlusal readjustment visits took place with the following periodicity: one week, two weeks, and one month after the beginning of treatment, and thereafter, every month. The control group was made up of patients who were treated with bite splint therapy only. For groups BS + B and BS + HS, in addition to the bite splint, the following protocol was followed: after disinfection of the pre-auricular area, they were injected with 1 mL of articaine (40 mg/mL) and adrenaline (10 µg/mL). A 23-gauge needle was used to inject 1 mL of betamethasone (Diprofos Depot 7 mg/mL) and 1 mL of sodium hyaluronate (Hyalart 10 mg/mL). The exact puncture point was determined by drawing the canthal-tragus line and measuring 10 mm from the tragus and 2 mm below the line. The zygomatic arch was palpated and the patients were asked to open their mouths to move the condyle anteriorly. The position of the needle should be from the outside in, from top to bottom and from back to front. Patients were evaluated one week after the beginning of the treatment, one month later and six months later. They were informed that they could experience discomfort in the region. No analgesic or anti-inflammatory drugs were prescribed. Throughout the process, all patients were always followed by the same practitioner. On the other hand, after signing the informed consent, the patients were randomly assigned to the respective treatment group. Treatment for each patient was assigned by a randomization list automatically generated prior to the start of the study in which the treatment approach was determined. In the BS + PRP group, the injections were preceded by the collection of the patient's peripheral blood from the ulnar vein into a glass tube with sodium citrate as an anticoagulant. After mixing the blood with the citrate, using rotating movements, the tubes were centrifuged at 3200 rpm for 12 min. After careful aspiration of platelet-rich plasma into a syringe, 2mL of PRP were injected into the TMJ following the previously described procedure for BS + B and BS + HS injections.

### 2.4. Pain and Mouth Opening Measurements

Pain was evaluated as previously described using a visual analogue scale [27]. In brief, this method consisted of a 10 cm line that represented the continuous spectrum of the painful experience: the left end indicated “no pain” and the right indicated the “the worst pain imaginable”. On this scale, patients indicated the degree of pain they experienced. Values ranged from 0 to 10, where 0 was no pain, 1–3 a little, 4–7 much and 8–10 unbearable.

To assess the pain-free mouth opening variable, a ruler was used to measure the distance between the patients’ incisors and the results were entered into an Excel datasheet.

### 2.5. Statistics

The descriptive statistics of the demographic data were carried out according to the groups studied. Gender was described in terms of absolute and relative frequency. Age was described using the mean and the standard deviation from the mean. Differences between groups were assessed using a three-way repeated measures ANOVA. The measures were time, the repeated factor, and the group defining the type of therapy and the gender were the other two factors. Differences at the initial moment were assessed using one-way ANOVA. The rates of change of pain and mouth opening were evaluated using linear regressions that were fitted in each group. The independent variable was time and this was expressed in weeks. In one case, pain was used as the dependent variable whereas pain-free mouth opening was used in the other case. As the slope (regression coefficient) of each linear regression represented the rate of change, the comparison between groups was direct and their confidence intervals (CI) allowed the assessment of statistical significance by considering the lack of overlapping between CIs.

The statistical analyses were carried out on the IBM SPSS^®^ v24 platform (Armonk, NY, USA) and a significance level of 0.05 was adopted.

## 3. Results

### 3.1. Patient Description 

The average age of the sample population was of 43.1 (SD 17.7) and the sex proportions were 20% for men and 80% for women. Sex distribution and age in the treatment groups yielded no significant differences. Supplementary Table 1 shows these data (Table 1).

### 3.2. Treatments Reduce Pain

**Figure 1:**
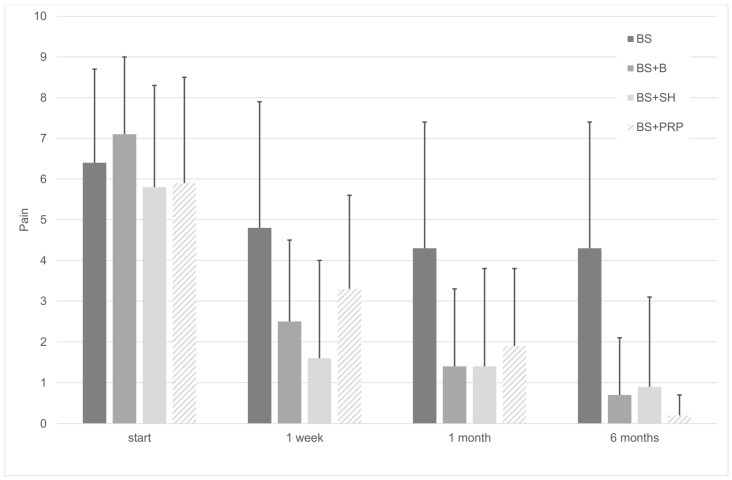
Pain change over time. Pain was reduced with the treatments (F(1.96, 71) = 175.880; *p* < 0.001).

There were no differences in pain intensity among the groups at the beginning of the study (F(3, 76) = 1.174; *p* = 0.325). Figure 1 shows values of pain intensity initially, 1 week, 1 month and 6 months after intervention. However, we found that after beginning therapy, there was a highly significant pain reduction (F(1.96, 71) = 175.9); *p* < 0.001), but there was no statistical evidence to establish differences between groups (F(3, 71) = 2.376; *p* = 0.077) and gender (F(1, 71) = 2.042; *p* = 0.257). Regression analysis evaluating pain variation over time showed that pain decreased significantly in all the groups treated with injections (*p* < 0.001), a decrease that was lower in the bite splint group with an average rate of 0.05 on the pain scale per week, and higher in the splint + PRP group, with an average rate of 0.172 per week. Results of the regression analyses regarding the pain are shown in Table 2.

### 3.3. Treatments Increase Mouth Opening

**Figure 2:**
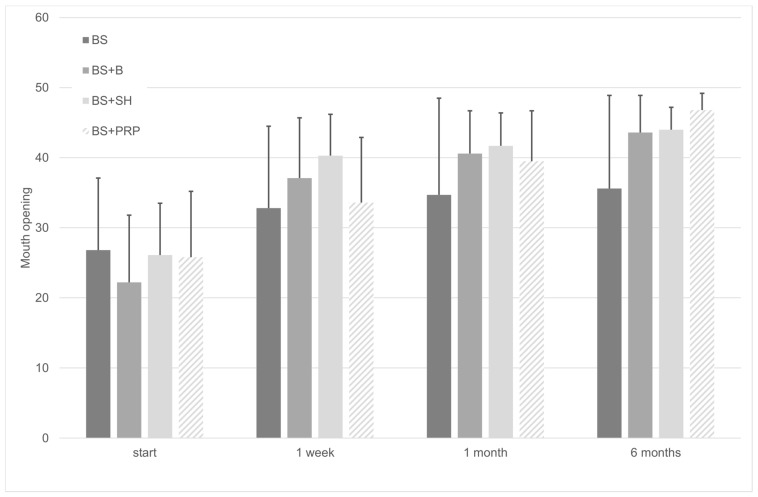
Mouth opening change over time. Pain-free mouth opening was increased with the treatments (F(1.99, 71) = 107.017), *p* < 0.001.

Variations in the patient’s maximum pain-free mouth opening over time with the different treatments were then analyzed (Figure 2). At the beginning of the treatment, there were no significant differences regarding mouth opening capacity (F(3, 76) = 0.995; *p* = 0.400). Subsequently, according to our linear regression analysis and ANOVA, pain-free mouth opening significantly increased over time in all four groups (F(1.99, 71) = 107.017), *p* < 0.001). The bite splint group had a smaller average rate of 0.219 per week, whereas the splint + PRP group had a larger average rate of 0.676 per week, as shown by the regression coefficients (B) in Table 3. Based on these data and according to this study, we can confirm that all four treatments succeeded in reducing pain intensity and improving mouth opening over time.

## 4. Discussion

The aim of this research was to assess the effectiveness of treatment with intra-articular injections of betamethasone, hyaluronic acid and platelet-rich plasma in combination with the nocturnal use of a bite splint compared with the isolated use of the splint.

In the current literature, there are several studies that have compared the outcome of the use of hyaluronic acid and corticosteroids [16,28,29,30,31,32], but very few have focused on the benefits of the use of platelet-rich plasma [33,34,35]. To the authors’ knowledge, there is no research currently available that compares the use of these three products in TMJ arthralgia.

It should be noted that, as demonstrated in several previous studies, the use of bite splint shows a high efficacy [36], as well as the use of intra-articular infiltrations of betamethasone, hyaluronic acid and platelet-rich plasma [28,29,31,32,33,34,35,37,38]. However, most of the studies associate them with more invasive procedures, such as arthrocentesis that is very effective in the TMJDs and can be performed on an outpatient basis under local anesthesia. These procedures also do not require elaborate and expensive equipment for their use [16,17,21,22,23,24,28,29,33]. However, we were looking for a more conservative but equally effective approach to the same problems and these were pointed out during this research. 

Treatment with a bite splint is one of the four therapeutic approaches included in this work and is also the one most commonly used, for the longest time around the world. It is known that in patients with temporomandibular disorders (not only of joint type, but also of muscle type), the vast majority of patients present a good response to the treatment, but there was a minority, no less important, that had no response [36]. Thus, for those patients, we had to choose another kind of treatment that is usually more invasive in order to improve their conditions. Some options are the intra-articular injections studied in this work. 

In this study, the group that only used the bite splint was the one that showed the least improvement compared to their initial condition in terms of maximum pain-free mouth opening and reduction in pain intensity. Regarding pain, values started at an average of 6.4 and ended with 4.3 after six months, representing an average decrease of 0.05 per week. The most marked period of pain reduction that took place was within the first week, an average value of 4.8. Regarding mouth opening, the results were very similar to those obtained for pain intensity, ranging from an average value of 26.8 mm and ending with 35.6 mm. Thus, a 6 mm increase in the mean was achieved at the end of the first week.

The fact that the bite splint group’s model has not been statistically significant could be due to the large dispersion of observed values.

In other studies, it has been reported that both intra-articular corticosteroid and sodium hyaluronate injections have a potent anti-inflammatory effect on synovial tissue and are known to reduce effusion, decrease pain and increase the range of motion of the synovial joints [29,32].

In this work, we found that treatment with sodium hyaluronate and betamethasone reported similar effectiveness. In terms of pain intensity, the patients initially reported an average of 5.8 and 7.1 for sodium hyaluronate and betamethasone use, respectively. The final values were 0.7 and 0.9, respectively, with both groups experiencing a drastic decrease in pain over the first week. Similarly, maximum pain-free mouth opening values started at 26.1 for sodium hyaluronate and at 22.2 for betamethasone, and after six months, very close values of 44 mm and 43.6 mm were reached, which are within normal opening values. 

Of note, PRP injections are widely used to treat different diseases in other joints of the human body due to their analgesic, anti-inflammatory and healing properties. However, there is a lack of studies which show these forms of treatment being used on TMJP [35]. 

First of all, it is important to mention that there are few studies involving the use of platelet-rich plasma in the treatment of TMJ arthralgia followed or not by arthrocentesis. Our results revealed an improved response to BS + PRP treatment compared to the previously commented therapies. A recent narrative review shows encouraging results for the use of platelet-rich plasma against other therapeutic approaches [33].

In the present study, the group that received platelet-rich plasma intra-articular injections benefited the most of the four groups studied, starting from pre-treatment pain intensity values of 5.9 and obtaining mean values of 0.2 after six months of treatment. This means that values decreased by 0.172 each week. Patients also started with maximum pain-free mouth opening values of 25.8 mm and these improved to 46.8 mm. However, it should be noted that, although there was a decrease in pain intensity over the first week to a value of 3.3 and an increase in pain-free mouth opening to 33.6 mm, these values fall behind those obtained for sodium hyaluronate and betamethasone.

## 5. Conclusions

We can conclude that all the therapeutic approaches in this study lead to significant improvements both in maximum pain-free mouth opening and in pain intensity reduction. The treatment with platelet-rich plasma and bite splint yielded the greatest results after six months. However, further study on the topic and new experimental designs are required to achieve a greater understanding of these results and to improve treatments.

## Figures and Tables

**Table 1:** Patient description.

Treatment	BS	BS + B	BS + SH	BS + PSP
Gender (M/F)	4/16 (20%/80%)	4/16 (20%/80%)	4/16 (20%/80%)	4/16 (20%/80%)
Age ($\bar{x}\;SD)$	41.3 (17.7)	40.8 (17.0)	37.4 (14.1)	36.7 (13.1)

BS: Bite splint; BS + B: Bite splint and betamethasone; BS + SH: Bite splint and sodium hyaluronate; BS + PRP: Bite splint and plasma-rich platelet.

**Table 2:** Regression analysis of pain over time.

	Pain Values				Regression Analysis		
Group	Start	1 Week	1 Month	6 Months	*p*	R^2^_adj_	B (IC95%)
BS	6.4 (2.3)	4.8 (3.1)	4.3 (3.1)	4.3 (3.1)	0.145 *	0.015	−0.050 (−0.118, 0.018)
BS + B	7.1 (1.9)	2.5 (2.0)	1.4 (1.9)	0.7 (1.4)	<0.001	0.240	−0.156 (−0.218, −0.094)
BS + SH	5.8 (2.5)	1.6 (2.4)	1.4 (2.4)	0.9 (2.2)	0.002	0.109	−0.108 (−0.174, −0.042)
BS + PRP	5.9 (2.6)	3.3 (2.3)	1.9 (1.9)	0.2 (0.5)	<0.001	0.343	−0.172 (−0.224, −0.119)

Data are shown as mean (SD). BS: Bite splint; BS + B: Bite splint and betamethasone; BS + SH: Bite splint and sodium hyaluronate; BS + PRP: Bite splint and plasma-rich platelet. R^2^_adj_: Adjusted coefficient of determination; B: regression coefficient *.

**Table 3:** Regression analysis of mouth opening over time.

	Mouth Opening Values (mm)				Regression Analysis		
Group	Start	1 Week	1 Month	6 Months	*p*	R^2^_adj_	B IC95%
BS	26.8 (10.3)	32.8 (11.7)	34.7 (13.8)	35.6 (13.3)	0.128	0.017	0.219 (−0.065, 0.503)
BS + B	22.2 (9.6)	37.1 (8.6)	40.6 (6.1)	43.6 (5.3)	<0.001	0.219	0.546 (0.316, 0.776)
BS + SH	26.1 (7.4)	40.3 (5.9)	41.7 (4.7)	44.0 (3.2)	<0.001	0.204	0.418 (0.237, 0.598)
BS + PRP	25.8 (9.4)	33.6 (9.3)	39.5 (7.2)	46.8 (2.4)	<0.001	0.372	0.676 (0.482, 0.871)

Data are shown as mean (SD) in mm. BS: Bite splint; BS + B: Bite splint and betamethasone; BS + SH: Bite splint and sodium hyaluronate; BS + PRP: Bite splint and plasma-rich platelet. R^2^_adj_: Adjusted coefficient of determination; B: regression coefficient.
